# A pseudo-R^2 ^measure for selecting genomic markers with crossing hazards functions

**DOI:** 10.1186/1471-2288-11-28

**Published:** 2011-03-15

**Authors:** Sigrid Rouam, Thierry Moreau, Philippe Broët

**Affiliations:** 1 Genome Institute of Singapore, Biopolis, Singapore; 2 Univ Paris-Sud, U669, Villejuif, F-94807 France; 3 Inserm, UMRS 1018, Villejuif, F-94807 France; Univ Paris-Sud, Villejuif, F-94807 France; 4 Hôspital Paul Brousse AP-HP, Villejuif, F-94807 France

## Abstract

**Background:**

In genomic medical studies, one of the major objectives is to identify genomic factors with a prognostic impact on time-to-event outcomes so as to provide new insights into the disease process. Selection usually relies on statistical univariate indices based on the Cox model. Such model assumes proportional hazards (PH) which is unlikely to hold for each genomic marker.

**Methods:**

In this paper, we introduce a novel pseudo-R^2 ^measure derived from a crossing hazards model and designed for the selection of markers with crossing effects. The proposed index is related to the score statistic and quantifies the extent of a genomic factor to separate patients according to their survival times and marker measurements. We also show the importance of considering genomic markers with crossing effects as they potentially reflect the complex interplay between markers belonging to the same pathway.

**Results:**

Simulations show that our index is not affected by the censoring and the sample size of the study. It also performs better than classical indices under the crossing hazards assumption. The practical use of our index is illustrated in a lung cancer study. The use of the proposed pseudo-R^2 ^allows the identification of cell-cycle dependent genes not identified when relying on the PH assumption.

**Conclusions:**

The proposed index is a novel and promising tool for selecting markers with crossing hazards effects.

## Background

In genomic medical research, one of the major objectives is to identify genomic markers having a prognostic impact on clinical outcomes (e.g. relapse, death) so as to provide new insights into the disease process. Most of the studies which investigate the relationship between genomic markers and time-to-event outcomes usually rely on marginal survival analysis that consider univariate prognostic indices derived from the semi-parametric Cox proportional hazards model. This proportional hazards (PH) assumption states that the ratio of the hazard functions of different individuals remains constant over time. Although this assumption is arbitrary, it is widely used since it offers a convenient way to summarize the effect of a covariate on the baseline hazard function and the resulting inference on the parameters of the model is robust enough to encompass some instances of non-proportionality (monotone, converging or diverging hazard functions). However, this PH modelisation is clearly not coping with crossing hazard functions. Crossing-hazards models explicitly specify that there is a time at which the hazard curves for different levels of a covariate cross. To our best knowledge, the crossing hazards phenomenon is barely investigated in genomic studies and it is usually described as a time-dependent effect of the genomic marker without any meaningful bioclinical interpretation.

In this paper, we introduce a novel pseudo-R^2 ^index derived from a semi-parametric non-proportional hazards model that is suited for the selection of genomic markers with crossing hazard functions. We also discuss one of the plausible interpretations for such crossing phenomenon that relates to a gene effect modification. For censored survival data, two main sapproaches have been considered for quantifying the predictive ability of a variable to separate patients: concordance and proportion of explained variation. This latter quantifies the relative gain in prediction ability between a covariate-based model and a null model, by analogy with the well-known linear model (and the R^2 ^criterion). In this framework, we propose a novel statistical quantity which is related to the score statistic. The proposed pseudo-R^2 ^index relies on the partial likelihood function in such a way that it has an interpretation in terms of percentage of separability between patients according to their survival times and marker measurements. It extends a previous work [1] for taking into account crossing hazards situations. From a real example, we show that the proposed index can be used to select genes with crossing hazards behavior that potentially indicate a modification of their prognostic effect. Moreover, it proves useful for identifying genomic markers with a common effect across multiple genomic studies due to its weak dependence on sample size variations. The paper is organized as follows. In section Methods, we first introduce an example of a simple interplay between two markers (effect modification) that leads to marginal crossing hazard functions and has prompted us to derive a novel pseudo-R^2 ^measure for such non-proportional situations. Then, we introduce a semi-parametric non-proportional hazards model which gives rise to some crossing effect of the hazard function. Finally, we derive from this model a pseudo-R^2 ^measure well-suited for crossing hazard function and show its link to the robust score statistic for testing no effect of the considered marker [2]. In the Results section, we report and discuss the properties of the index from simulations experiments and compare them to those of classical indices [3-7] which are also linked to the likelihood function. In the Example section, we illustrate the use of the index for selecting genomic factors with crossing hazard effect in a lung cancer study. In the last section, we summarize our work.

## Methods

In this section, we first present a simple situation which motivates the use of the semi-parametric non-proportional hazards model introduced in the next subsection.

### Notations

Let the random variables *X *and *C *be the failure and censoring times, and *T *= min(*X*, *C*) be the observed follow-up time. The random variables *T *and *C *are assumed to satisfy the condition of independent censoring [8]. We denote {*N_i_*(*t*), *t *≥ 0} the counting process that indicates the number of events that have occurred in the interval (0, *t*] for subject *i*, *i *= 1,⋯, *n*, so that *N_i_*(*t*) takes values 0 or 1. Let *Y_i _*be the at-risk process, so that *Y_i_*(*t*) = 1 indicates that subject *i *is at risk just before time *t*, and *Y_i_*(*t*) = 0 otherwise.

Let *dN_i_*(*t*) = *N_i_*(*t*^- ^+ *dt*) - *N_i_*(*t*^-^) be the number of events occurring in the interval [*t*, *t *+ *dt*) for subject *i*,  the total number of events that have occurred in the interval (0, *t*] and  the number of subjects at risk at time t. Finally, let  represent the value of the g^th ^covariate for individual *i*.

### Motivational situation: the modulating effect

In the following, we show how a simple interplay between two binary markers *Z*^(1) ^and *Z*^(2) ^can lead to marginal crossing hazard functions.

The joint distribution of *Z*^(1) ^and *Z*^(2) ^is defined by:

It is assumed that the hazard function of subject *i *with  and  is given by(1)

where *λ*_0_(*t*) is an arbitrary unspecified baseline hazard function, and *α *and *γ *are unknown regression coefficients.

Model (1) describes a modulating effect of the two markers *Z*^(1) ^and *Z*^(2)^, whereby *Z*^(2) ^has a multiplicative effect on the hazard and *Z*^(1) ^has a multiplicative effect only if *Z*^(2) ^equals one (so called effect modification). The corresponding hazard functions according to the values of *Z*^(1) ^and *Z*^(2) ^are shown in Table 1.

**Table 1:** Hazard function

	*Z*^(1)^	
	
*Z*^(2)^	0	1
0	*λ*_0_(*t*)	*λ*_0_(*t*)
1	*λ*_0_(*t*)*e*^γ^	*λ*_0_(*t*)*e*^α+γ^

Assuming that model (1) is the true one, the consequences of omitting *Z*^(2) ^on the formulation of the observed hazards ratio relative to *Z*^(1) ^is described below. Expressing model (1) in terms of the conditional survival function given  leads to:

where *S*_0_(*t*) is the survival function corresponding to the baseline hazard function *λ*_0_(*t*). The survival function given () follows directly from Bayes' theorem, and the hazard function given () can be easily deduced as:

It is worth noting that this latter expression can be obtained as the expectation of (1) taken over *Z*^(2) ^given the at risk process. Finally, the hazards ratio relative to the values  and  is given by:(2)

It appears from this expression that hazards may cross over time. More precisely, it is shown in Additional File 1 that when *α *and *γ *are positive and assuming a balanced joint distribution for (*Z*^(1)^, *Z*^(2)^), the hazards ratio inverts at a given time in (0; +∞). Obviously, such a time-dependence cannot be properly handled by using the proportional hazards model to analyze the data.

### Semi-parametric model

The proposed model defines the survival function of subject *i *with covariate *Z_i _*as follows(3)

where *λ*_0_(*t*) is an unspecified baseline hazard function and *β *an unknown regression parameter. It is a particular case of a model that was proposed for handling hazards ratio that invert over time [9], and it corresponds to a semi-parametric generalization of the Weibull distribution. For subject *i*; *i *= 1, ⋯, *n*, the model (3) can be written in terms of the hazard function(4)

where  is the cumulative baseline cumulative hazard function.

In the simple case of a covariate *Z *taking values 0 or 1, the hazards ratio (HR) of the two groups corresponding to *Z *= 1 and *Z *= 0 is equal to . If *β *> 0, this function is increasing from 0 to +∞ and takes value 1 for . In this case, the risk of occurrence of the event is smaller in group 1 than in group 0 for 0 ≤ *t *< τ, and becomes greater when *t *> τ. If *β *< 0, the hazards ratio is decreasing from +∞ to 0 and takes value 1 for *t *= τ as calculated above. The risk of occurrence of the event is thus greater in group 1 than in group 0 when 0 <*t *≤ τ and becomes smaller for *t *> τ.

Thus, as expected, model (4) allows hazards to cross over time. Note that the survival functions cross at a time larger than the crossing time of the hazards, and may not cross at a finite time.

At time *t *and for an individual *i*, *i *= 1, ⋯, *n*, the first derivative of the partial log-likelihood with respect to *β *is the score function:

The score function evaluated for *β *= 0 can be written at time *t *as(5)

with *ω*(*s*) = 1 + log{Λ_0_(*s*)}.

### Pseudo-R^2 ^measure

The goal of this section is to propose a pseudo-R^2 ^index that can be interpreted in terms of percentage of separability between patients according to their survival times and marker measurements under the crossing hazards model (4). The approach used below is based on the score function (5). It extends the particular case that we considered in a former work [1] where we assumed the classical PH model. The main idea is to note that the score can be rewritten as a separability quantity between patients experiencing or not the event of interest. More precisely, the quantities *U_i _*in (5) can be rewritten as

With .

From this expression, we show that, for a given covariate *Z *at time *t*, the *U_i _*can be expressed as the weighted difference between the value of the covariate of the patient observed to experience the event of interest and the mean of the covariates of the group of patients observed to not experience the event.

An estimation of the *U_i _*is given by

where , Λ_0_(*t_i_*) is estimated by the left-continuous version of the Nelson's estimator [10,11], and *δ_i_*, *i *= 1, ⋯, *n *is the indicator of failure at time *t_i_*.

For distributional reason, instead of the *U_i_*, we introduce the so called robust scores *W_i _*[2] which expressions are(6)

Where

The *W_i _*can be estimated by(7)

The sum over *i *of the robust *W_i_*, *i *= 1, ⋯, *n *is identical to the sum of the *U_i_*. However, as for the Cox model, the *W_i _*are independent, while the *U_i _*are not.

Finally, the index is equal to the robust score statistic divided by the number of distinct uncensored failure times *k*:

The index **D**_0 _is interpreted in terms of percentage of separability over time between the event/non-event groups. Its calculation is easy as it does not require the estimation of the parameter *β *of the crossing hazards model. We can easily demonstrate that 0 ≤ **D**_0 _≤ 1.

It is worth noting that the index **D**_0 _can be interpreted as a pseudo-R^2 ^measure. In the linear regression model, the R^2 ^(coefficient of determination) can be directly linked to likelihood-related quantities such as the Wald test, the likelihood ratio and the score statistics (see [12]). These formal relationships provide different ways to interpret the R^2^. In the framework of non-linear models, statisticians have searched for a corresponding index and different pseudo-R^2 ^statistics have been proposed for censored data. Our proposed index is an extension of the definition of the R^2 ^for survival model with crossing hazards which relies on the score statistic.

## Results

### Simulation Scheme

A simulation study was performed to describe the behavior of the proposed index, , in finite samples generated under the crossing hazards model (3), and to compare it to the behavior of other existing indices. Different situations were considered, corresponding to different covariate distributions, regression parameter values and sample sizes. The influence of various censoring distributions was also investigated. The indices that were compared to  include our previous index derived from a PH model (i.e. calculated according to the same approach than  with *ω*(*t_i_*) = 1, [1]) and other most usual indices: Allison's index [3], its modified version [5], Nagelkerke [4] and Xu and O'Quigley's [6] indices. All of them are designed for a PH model and are denoted ,  and , respectively. The different elements defining a configuration were the following. For a given subject, the distribution of the covariate *Z *included in model (3) was either discrete (Bernoulli ℬ(0.5)) or continuous (log-normal with mean 0 and variance 1/4, or uniform . These three distributions of *Z *were standardized to have the same variance. Two distributions for the survival time *X *were considered. The first one was defined by model (3) with Λ_0_(*t*) = *t*. It is equivalent to a Weibull parametric model  with scale parameter *η *= 1 and shape parameter *α *= exp(*βZ*). The second one correspond to a log-normal distribution with mean equal to 0 and standard deviation equal to *e^-βZ^*. These two distributions allowed to simulate the crossing hazards phenomenon.

The coefficient *β *was given a value such that *e^β ^*= 1, or 2, or 3, or 4. It can be noticed that, in the case of a Bernoulli variable *Z*, the hazard functions corresponding to *e^β ^*= 2, or 3, or 4 cross when the survival function of group *Z *= 1 equal 0.78, 0.82, 0.85 respectively. The censoring variable *C_i _*was assumed to be independent from *X_i _*given *Z_i _*and distributed according to either a uniform  or exponential *C_i _*~ℰ(*γ*) distribution. The parameters *r *and *γ *were calculated in order to yield an expected overall percentage of censoring *p_c _*equal to 0%, 25% and 50%. The sample size *n *was taken equal to 50, 100 or 500. Data were generated as follows. For each subject *i*, *i *= 1, ⋯, *n*, a value of the covariate *Z_i _*was generated. Given that value, a survival time *X_i _*was generated according to a either Weibull distribution , or a log-normal distribution . The censoring variable *C_i _*was independently generated and the observed follow-up time *T_i _*was calculated as min(*X_i_*, *C_i_*). For each configuration 1,000 independent replications were generated.

### Simulation Results

**Figure 1:**
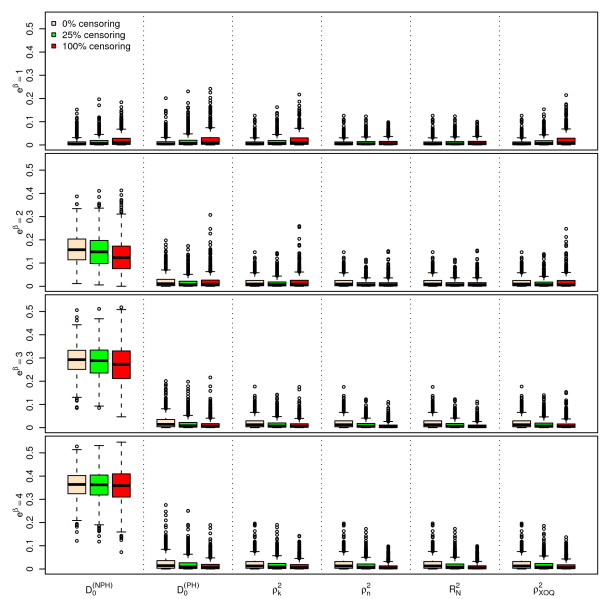
**Simulations results for ****, ****, **** and ****, for n = 100 subjects, **** and a uniform censoring (1,000 repetitions)**. Boxplots of the different indices according to the values of *e*^*β *^and *p*_*c*_.

**Figure 2:**
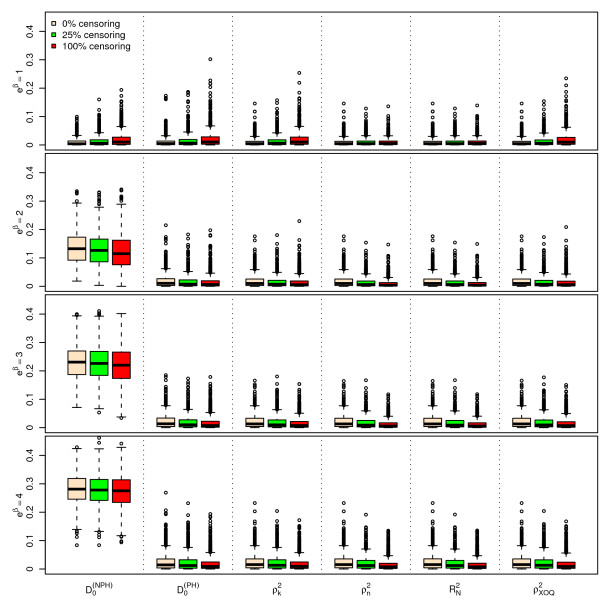
**Simulations results for ****, ****, **** and ****, for n = 100 subjects, **** and a uniform censoring (1,000 repetitions)**. Boxplots of the different indices according to the values of *e*^*β *^and *p*_*c*_.

**Figure 3:**
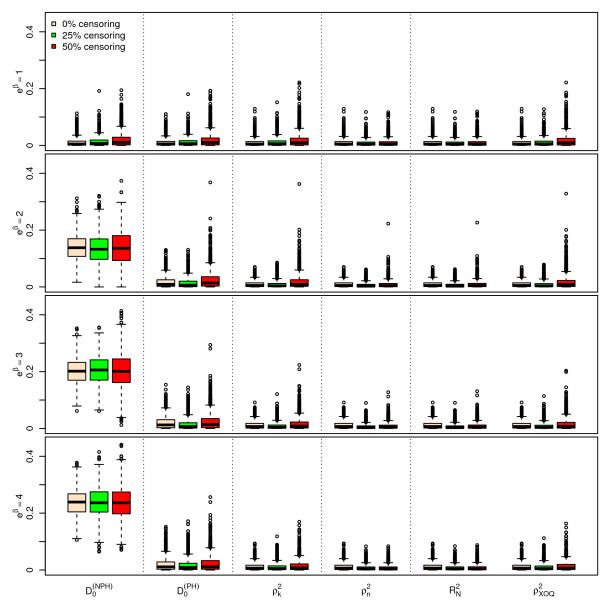
**Simulations results for ****, ****, **** and **** and ****, for n = 100 subjects, **** and a uniform censoring (1,000 repetitions)**. Boxplots of the different indices according to the values of *e*^*β *^and *p*_*c*_.

**Figure 4:**
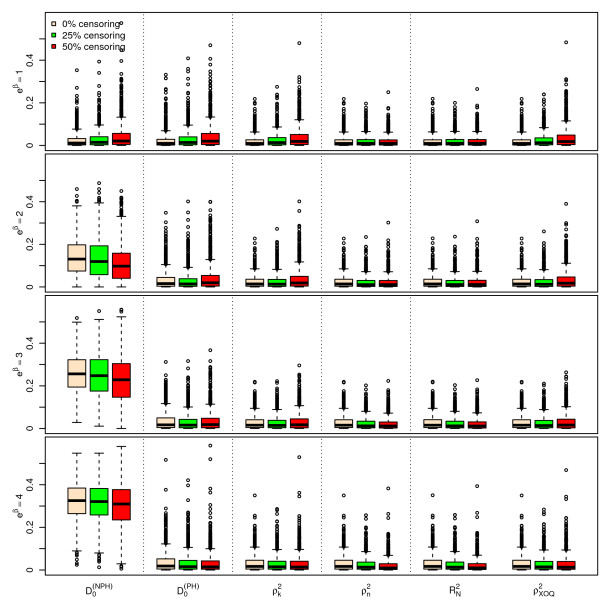
**Simulations results for ****, ****, **** and ****, for n = 50 subjects, **** and a uniform censoring (1,000 repetitions)**. Boxplots of the different indices according to the values of *e*^*β *^and *p*_*c*_.

**Figure 5:**
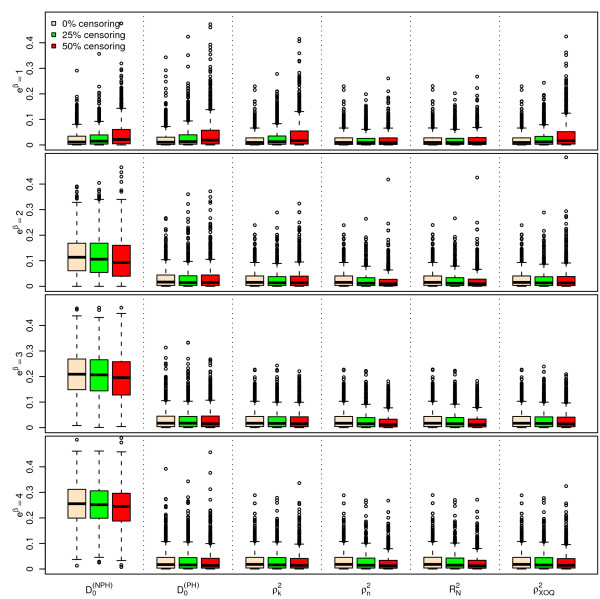
**Simulations results for ****, ****, **** and ****, for n = 50 subjects, **** and a uniform censoring (1,000 repetitions)**. Boxplots of the different indices according to the values of *e*^*β *^and *p*_*c*_.

**Figure 6:**
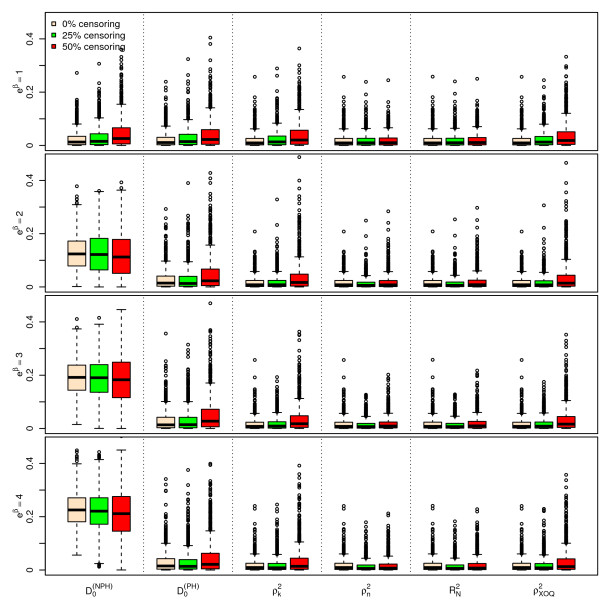
**Simulations results for ****, ****, **** and ****, for n = 50 subjects, **** and a uniform censoring (1,000 repetitions)**. Boxplots of the different indices according to the values of *e*^*β *^and *p*_*c*_.

Figures 1, 2 and 3 display the results of the simulations obtained for , ,  and  for *n *= 100, for a Bernoulli, a uniform and a log-normal distribution, respectively, according to the values of *e^β ^*and the percentage of censoring *p_c_*. Figures 4, 5 and 6 give the results for *n *= 50. The results for *n *= 500 are given in Additional Files 2, 3 and 4.

As seen from Figures 1, 2, 3, 4, 5 and 6 and Additional Files 2, 3 and 4, when *β *= 0, i.e. in the absence of covariates, the different indices are close to zero for *n *= 50, 100 and 500. Among the six indices, only  shows a mean value increasing regularly with *β*. The means of the five other indices do not appear to increase with *β *and remain below 0.05 even for the highest value of *e^β ^*= 4. When *β ≠ *0, the mean values of  for the different sample sizes are fairly stable.

The standard errors of the six indices are small when *β *= 0. The standard errors of  are larger than those of the other indices when *β *≠ 0 and slightly decrease as *β *increases. As expected, the standard errors of the different indices decrease when *n *increases.

The mean value of  does not appear to be sensitive to the censoring rate. However, the standard error of this index moderately increases as the percentage of censoring increases from 0% to 50%. In addition, the results obtained with a log-normal distribution of the survival time *X *on one hand (see Additional Files 5, 6 and 7 for the case n = 100), and with an exponential distribution of the censoring variable, on the other hand (not shown) are very similar, concerning  and the other indices as well.

### Application of the index on real data

In this section, we illustrate the use of the proposed index by selecting transcriptomic prognostic factors having a crossing effect in a lung cancer study. We compare the selection to the one obtained when relying on the index calculated under a proportional hazards model.

### Dataset

This series is composed of 74 patients who underwent surgery at the Hôtel-Dieu Hospital (AP-HP, France) between August 2000 and February 2004 for stage IB (pT2N0) primary adenocarcinoma or large cell lung carcinoma of peripheral location [13]. Relapse-free survival was defined as the time from surgery until disease-related death, disease recurrence (either local or distant), or last follow-up examination. The median relapse-free survival time was 63.8 months. The two years relapse-free survival was 80.3%[71.2%, 90.5%], and the five years relapse-free survival was 59.3%[47.2%, 74.5%]. For each patient, we considered the gene expression measurments of 51,852 transcripts (obtained using Aymetrix HU133 Plus 2.0; Aymetrix, Santa Clara, CA, USA) located on the autosomal chromosomes.

### Selection of the variables

The genes were ranked according to the value of either  or . We decided to focus our attention on the first 200 top-ranked transcripts in both cases. The lowest separability for both indices were close to each other (29.8% for  and 29.1% for ). Only a small proportion of transcripts (5%) was common to both lists.

We then examined the biological processes that were significantly over-represented in the two sublists using the PANTHER (Protein ANalysis THrough Evolutionary Relationships) classification system [14]. Results showed that the two indices allow selecting genes involved in different biological processes (See Additional File 8). For the cell-cycle process,  selected 25 transcripts (significantly higher number than the 5% expected by chance), whereas  selected only 16 transcripts (not significantly higher number than the 5% expected by chance). The two lists of genes involved in cell cycle are given in Additional File 9.

**Figure 7:**
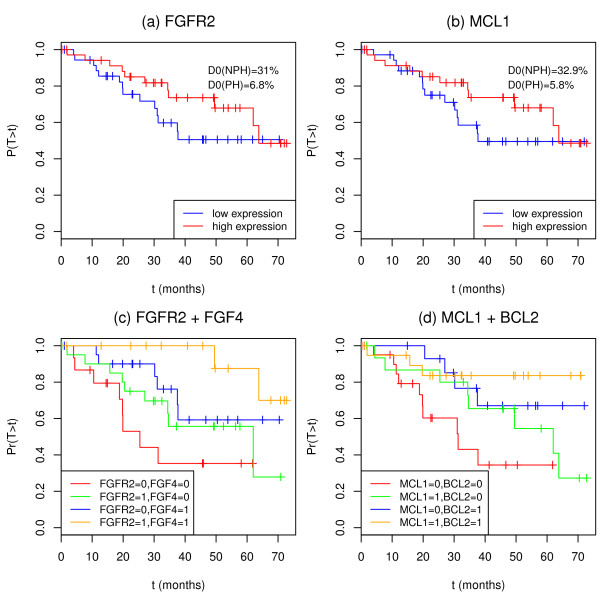
**Kaplan-Meier curve of the groups according to the expression levels of genes (a) *FGFR2 *, (b) *MCL1 *, and of the groups defined by the four combinations of expression levels of (c) *FGFR2 *and *FGF4 *, and (d) *MCL1 *and *BCL2 *in the lung cancer study**. The groups of patients were determined according to the low-high expression status of the genes considered. Patients whose expression measurement was higher (resp. lower) than the median were assigned to the "highly expressed" (resp. "lowly expressed") group.

Among the 25 cell-cycle related transcripts selected according to , we discussed the behavior of two genes, namely *FGFR2, MCL1*, known to be involved in complex biological pathways. In particular, we examined whether the crossing phenomenon (observed om Figures 7.a and 7.b) could potentially be related to some effect modification of other genes.

The gene *FGFR2 *(fibroblast growth factor receptor 2) is known to be involved in various cancer types [15] and low gene expression measurements have been reported as linked to a shorter survival in lung cancer [16]. The analysis of *FGFR2 *gene expression taking into account *FGF4 *gene expression, which is one of its ligand, suggested a potential modulating effect between the two genes. In the following, we reported the hazards ratio (HR) computed under the Cox PH model for the four groups resulting from dichotomizing the two variables at the median. We also displayed the Kaplan-Meier curves on Figure 7.c. As seen on this latter, patients with low expression (below the median) of both *FGFR2 *and *FGF4 *have the worst prognosis (reference group). When *FGFR2 *is highly expressed (above the median) and *FGF4 *lowly expressed, the survival is not significantly improved (HR = 0.532 [0.202, 1.399]). However, the over-expression of *FGF4 *significantly improve the survival (HR = 0.329 [0.112, 0.967]). Finally, patients with a high expression of *FGFR2 *and *FGF4 *have the best prognosis (HR = 0.103 [0.021, 0.516]).

In the same way, we discussed the interaction between *MCL1 *and *BCL2*, two anti-apoptotic genes belonging to the *BCL-2 *gene family. Considered initially as oncogenes, the prognostic impact of *BCL-2/MCL-1 *for various type of cancer is debated due to their dual function on cell death and cell proliferation (for a review, [17]). The anti-apoptotic effect is associated with resistance to chemotherapy, leading an adverse prognostic role in some cancers such as leukemia or advanced ovarian tumors. In contrast, the anti-proliferative activity of *MCL-1 *and *BCL-2 *is associated with a favorable prognosis effect in some early carcinomas, such as lung adenocarcinoma [18]. Moreover, the combined analysis of *MCL1 *and *BCL2 *gene expressions indicated a potential modulating effect between them. As seen on Figure 7.d., in our lung cancer study of early lung adenocarcinomas treated by surgery alone, patients with low expression (below the median) of both genes *MCL1 *and *BCL2 *have the worst prognosis (reference group). When *MCL1 *is highly expressed (above the median) and *BCL2 *lowly expressed, the prognosis is not significantly improved (HR = 0.533[0.202, 1.4103]). On the contrary, patients with low expression of *MCL1 *and high expression of *BCL2 *have a significantly improved survival (HR = 0.296[0.091, 0.962]). Finally, the over-expression of both *MCL1 *and *BCL2 *gives the best prognosis (HR = 0.189[0.051, 0.700]).

In these two examples, we could hypothesize that the crossing effect observed in the marginal analysis of *FGFR2 *or *MCL1 *is related to some potential effect modification linked to *FGF4 *or *BLC2*, respectively. This hypothesis is consistent with the known biological activity of those genes. *FGFR2 *encodes for a receptor, which needs one of its ligand (i.e. *FGF4 *) for activation and biological activity. Also, *BCL2 *and *MCL-1 *encode two proteins of the same family, which may act together on the cell through heterodimerization on apoptosis or cell proliferation. The resulting subgroup of patients defined by high expression of these genes couples might be clinically relevant and the object of further investigations.

## Discussion

For survival data analysis, univariate feature selection strategy is mainly based on ranking markers according to the value of a test statistic or a predictive index obtained under the classical Cox PH model. In such setting, we demonstrated in a previous work the interest of using a pseudo-R^2 ^measure for genomic studies. However, various departures from the PH assumption can be observed and crossing hazards phenomenon can be encountered in real situations.

In this context, we propose a novel pseudo-R^2 ^measure that is suitable for identifying genomic markers with crossing effects. It is linked to a semi-parametric survival model that provides sufficient flexibility to handle data with crossing hazards. Selecting such markers is potentially important since it could reflect the complex interplay between genes belonging to the same pathway.

The proposed index is ranging from zero to one and can be interpreted in terms of percentage of separability over time between the subgroup of subject(s) experiencing the event and the subgroup of those experiencing the event at a later time. It quantifies the prognostic separability of markers under a crossing hazard function assumption, whereas for the proportional hazards setting other specialized indices have previously been proposed [1]. This pseudo-R^2 ^is derived from the partial log-likelihood function and directly linked to the robust score statistic, while similar derivations from Wald or likelihood ratio statistics are not trivial and not easily tractable. As seen from our simulation results, the proposed index increases with the value of the regression parameter and is affected neither by the percentage of censoring nor the sample size of the study. The results show that our pseudo-R^2 ^is the most suitable for taking into account the crossing hazards phenomenon, as compared to classical indices.

From a real dataset on lung cancer, we show that our index allows to identify genes involved in biological processes linked to the tumor evolution and that are not selected under the PH assumption.

Among the cell-cycle related genes of our selection, we investigate two genes, *FGFR2 *and *MCL1*, which crossing effects could potentially be linked to some modulating effect due to other genes from the same biological pathway. Knowing the complexity of gene interactions, this is an over-simplification of the biological reality and other mechanisms can obviously lead to such non-proportional phenomenon. In this analysis, the gene expression measurements are dichotomized based on the median but other cutoffs could be investigated (by searching for optimal cutoff point) as proposed by Motakis *et al*. [19]. To the best of our knowledge, the present work is the first to propose a pseudo-R^2 ^measure that is specifically designed for crossing hazards situations.

## Conclusions

We propose a novel pseudo-R^2 ^measure that quantifies the prognostic separability of markers under a crossing hazard function assumption. This phenomenon can be encountered in real situations promoting the use of this novel index.

## Competing interests

The authors declare that they have no competing interests.

## Authors' contributions

SR, TM and PB developed the original index. PB coordinated the project and is SR's PhD thesis advisor. All authors read and approved the final manuscript.

## Pre-publication history

The pre-publication history for this paper can be accessed here:

http://www.biomedcentral.com/1471-2288/11/28/prepub

## Supplementary Material

Additional file 1 **The hazards ratio function inverts for a given time**. Click here for file

Additional file 2**Simulations results for ****, ****, **** and ****, for n = 500, **** and a uniform censoring (1,000 repetitions)**. Graphic: Boxplots of the different indices according to the values of *e*^*β *^and *p*_*c*_.Click here for file

Additional file 3**Simulations results for ****, ****, **** and ****, for n = 500, **** and a uniform censoring (1,000 repetitions)**. Graphic: Boxplots of the different indices according to the values of *e*^*β *^and *p*_*c*_.Click here for file

Additional file 4**Simulations results for ****, ****, **** and ****, for n = 500, **** and a uniform censoring (1,000 repetitions)**. Graphic: Boxplots of the different indices according to the values of *e*^*β *^and *p*_*c*_.Click here for file

Additional file 5**Simulations results for ****, ****, **** and ****, for n = 100 subjects, **** and a uniform censoring (1,000 repetitions)**. Graphic: Boxplots of the different indices according to the values of *e*^*β *^and *p*_*c*_.Click here for file

Additional file 6**Simulations results for ****, ****, **** and ****, for n = 100 subjects, **** and a uniform censoring (1,000 repetitions)**. Graphic: Boxplots of the different indices according to the values of *e*^*β *^and *p*_*c*_.Click here for file

Additional file 7**Simulations results for ****, ****, **** and ****, for n = 100 subjects, **** and a uniform censoring (1,000 repetitions)**. Graphic: Boxplots of the different indices according to the values of *e*^*β *^and *p*_*c*_.Click here for file

Additional file 8**Biological processes (obtained from the PANTHER classification system) for the lung cancer study cohort**. Table: List of the biological process obtained according to  and .Click here for file

Additional file 9**Lists of the cell cycle related transcripts among the selection according to the value of the index calculated either under the crossing hazards effect or the PH model**. Tables: Lists of the cell cycle related transcripts according to  and .Click here for file
