# Supplementary material for: A pseudo-R2 measure for selecting genomic markers with crossing hazards functions
Source: BMC Med Res Methodol. 2011 Mar 15;11:28. doi: 10.1186/1471-2288-11-28 (PMC3068986; doi:10.1186/1471-2288-11-28)
Supplement: Additional file 1 — The hazards ratio function inverts for a given time. [file 1471-2288-11-28-S1.PDF]

Additional file 1: The hazards ratio function inverts for a given time.

The purpose is to show that, under certain conditions, the hazards ratio (HR) in equation (2),

$$HR(t) = \frac{\lambda(t|Z_i^{(1)} = 1)}{\lambda(t|Z_i^{(1)} = 0)} = \left(\frac{p_{11}e^{\alpha+\gamma}S_0(t)^{e^{\alpha+\gamma}} + p_{10}S_0(t)}{p_{11}S_0(t)^{e^{\alpha+\gamma}} + p_{10}S_0(t)}\right) \times \left(\frac{p_{01}S_0(t)^{e^{\gamma}} + p_{00}S_0(t)}{p_{01}e^{\gamma}S_0(t)^{e^{\gamma}} + p_{00}S_0(t)}\right)$$

equals one at a given time t_0 in $(0;+\infty)$, being greater than one for $t < t_0$ and lesser than or equal to one for $t > t_0$.

In the sequel, it is assumed that $p_{11} = p_{10} = p_{01} = p_{00}$ and that $\alpha > 0$, and $\gamma > 0$. The following notations will be used: $k = \alpha/\gamma$, $a = \exp(\gamma)$ with $a > 1$, and $X = S_0(t)$ where X increases in $(0, 1]$ as t decreases from $+\infty$ to 0. For $X > 0$, HR becomes:

$$\begin{aligned} HR(X) &= \left(\frac{a^{k+1}X^{(a^{k+1}-1)} + 1}{X^{(a^{k+1}-1)} + 1}\right)\left(\frac{X^{a-1} + 1}{aX^{a-1} + 1}\right) \\ &= \frac{a^{k+1}X^{(a^{k+1}+a-2)} + a^{k+1}X^{(a^{k+1}-1)} + X^{a-1} + 1}{aX^{(a^{k+1}+a-2)} + X^{(a^{k+1}-1)} + aX^{a-1} + 1} = \frac{N(X)}{D(X)} \end{aligned}$$

Note that if X tends towards zero (i.e. if t tends towards infinity), $HR(X)$ tends towards 1 (as does $HR(t)$). In order to prove the existence and uniqueness of $0 < X_0 < 1$ such that $HR(X_0) = 1$, it is useful to consider the difference $(N(X) - D(X))$. More precisely, noting :

$$N(X) - D(X) = f(X)X^{a-1} \quad (8)$$

$$\text{with } f(X) = (a^{k+1} - a)X^{(a^{k+1}-1)} + (a^{k+1} - 1)X^{a^{k+1}-a} + (1 - a) = 0.$$

It is obvious that the searched X_0 is the solution, if any, of the equation $f(X) = 0$.

The first derivative of f relative to X is equal to

$$\frac{\partial f(X)}{\partial X} = (a^{k+1} - a)(a^{k+1} - 1)\left[X^{(a^{k+1}-2)} + X^{a^{k+1}-a-1}\right]$$

It is positive on $(0; 1)$ since $a > 1$ and $k > 0$, so that f is increasing on $(0; 1)$. As $f(0) < 0$ and $f(1) > 0$, the equation $f(X) = 0$ has a unique solution X_0 on $(0; 1)$. Moreover, it follows that, for $0 < X < X_0$ ($X > X_0$, respectively), the function $f(X)$ is negative (positive, respectively) so that $(N(X) - D(X))$ is negative (positive, respectively) as shown by formula (8) above. Noting that $(N(X) - D(X))$ negative (positive, respectively) is equivalent to $HR(X) < 1$ (> 1, respectively), the above results can be summarized as follows (See also Table 1).

As expected, it exists an unique time value $t_0 = S_0^{-1}(X_0)$ with $0 < t_0 < +\infty$ such that $HR(t)$ is greater than one for $t < t_0$, and lesser than one for $t > t_0$. Note that the function $HR(t)$ is not monotone, since $HR(t)$ tends towards one as t tends towards $+\infty$, as already remarked.

Table 1: Summary of the signs of f and HR

X	0		X_0		1
$f(X)$	$1-a<0$	$-$	0	$+$	$2a^{k+1}-2a>0$
t	0		t_0		$+\infty$
$HR(t)$	$\frac{e^{\gamma(k+1)}+1}{e^{\gamma}+1}>1$	>1	1	<1	1^-
