# Supplementary figures and images for: A pseudo-R2 measure for selecting genomic markers with crossing hazards functions
Source: BMC Med Res Methodol. 2011 Mar 15;11:28. doi: 10.1186/1471-2288-11-28 (PMC3068986; doi:10.1186/1471-2288-11-28)

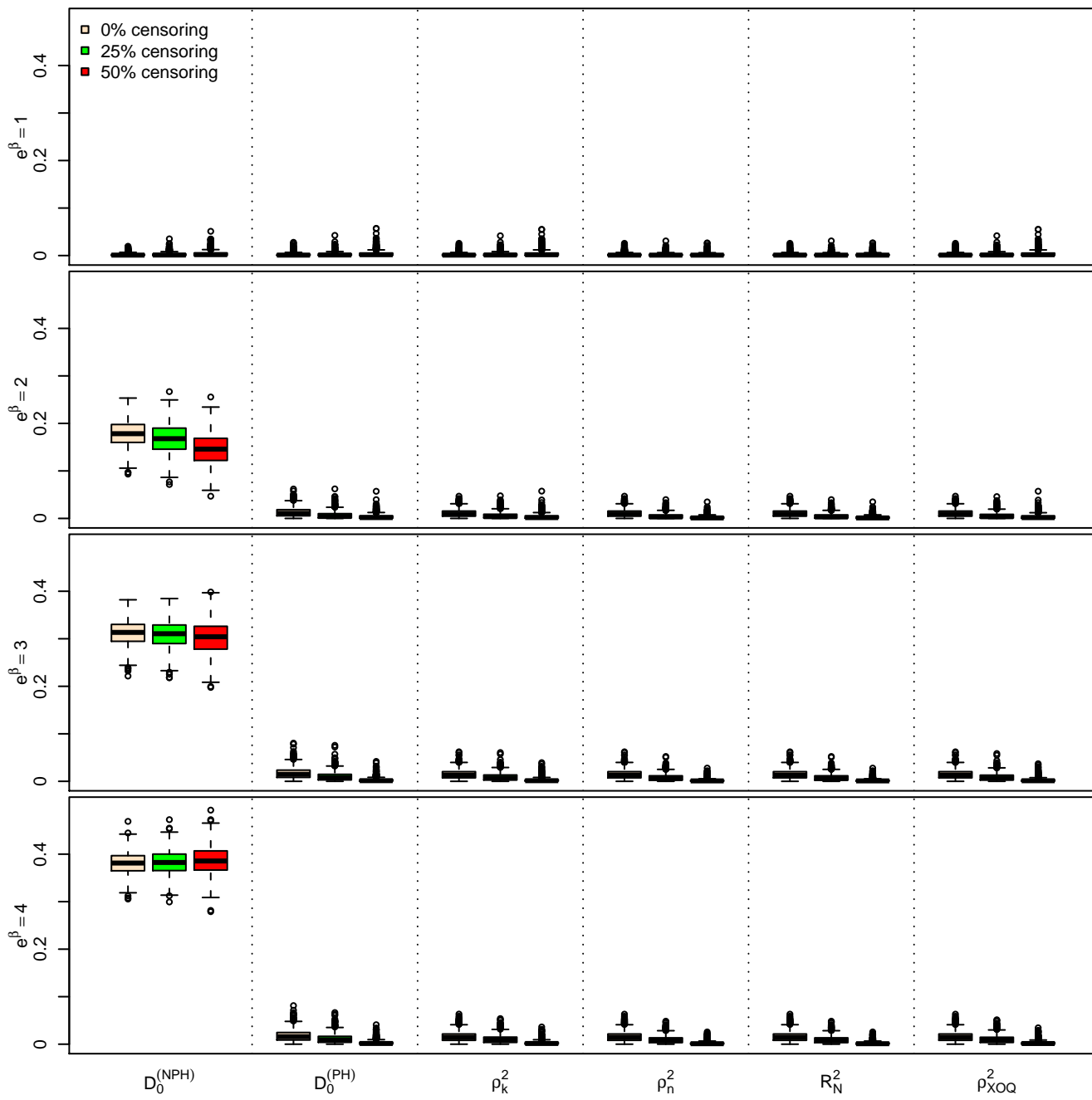

Supplement: Additional file 2 — Simulations results for , , and , for n = 500, and a uniform censoring (1,000 repetitions). Graphic: Boxplots of the different indices according to the values of eβ and pc. [file 1471-2288-11-28-S2.PDF]

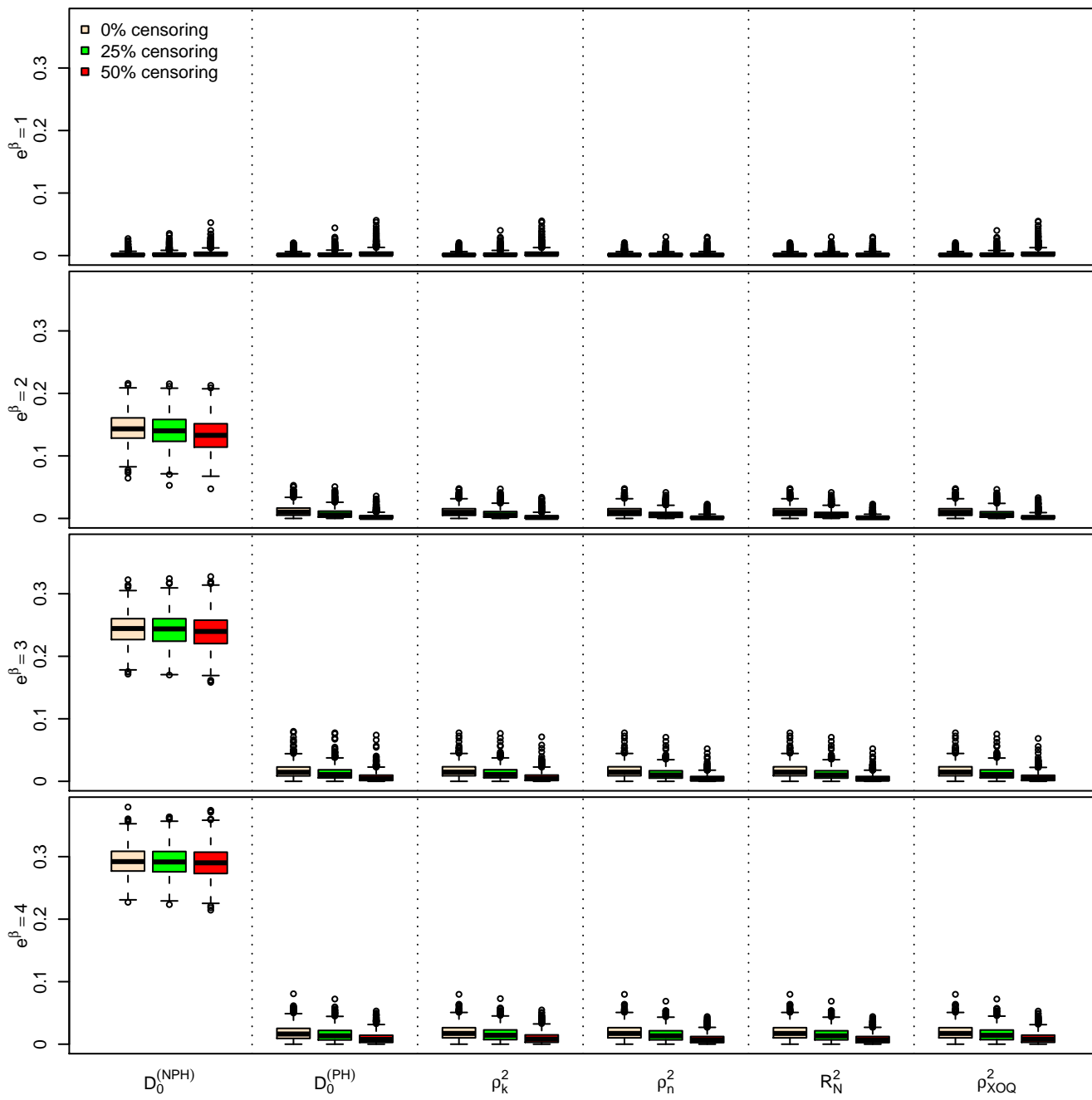

Supplement: Additional file 3 — Simulations results for , , and , for n = 500, and a uniform censoring (1,000 repetitions). Graphic: Boxplots of the different indices according to the values of eβ and pc. [file 1471-2288-11-28-S3.PDF]

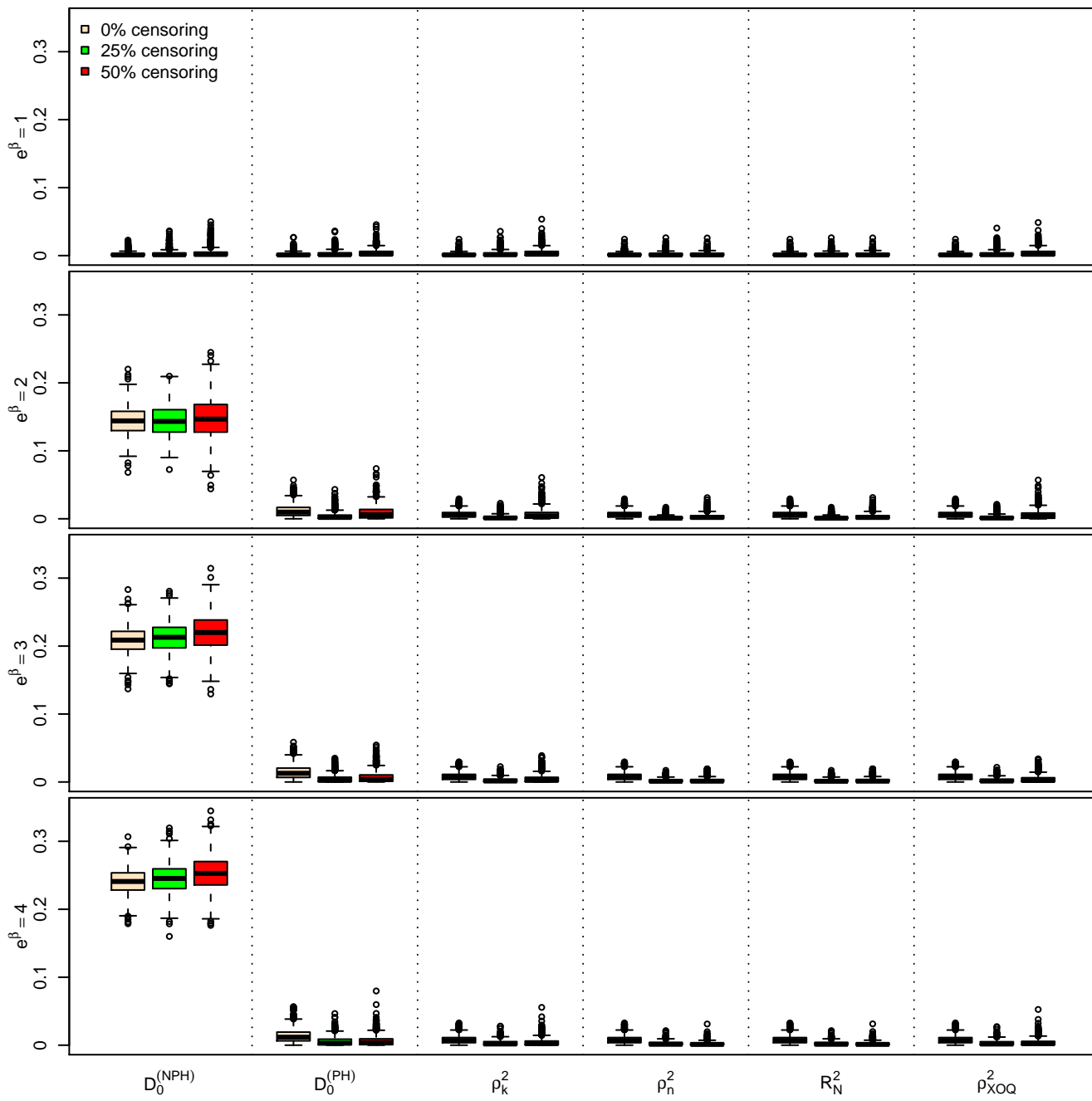

Supplement: Additional file 4 — Simulations results for , , and , for n = 500, and a uniform censoring (1,000 repetitions). Graphic: Boxplots of the different indices according to the values of eβ and pc. [file 1471-2288-11-28-S4.PDF]

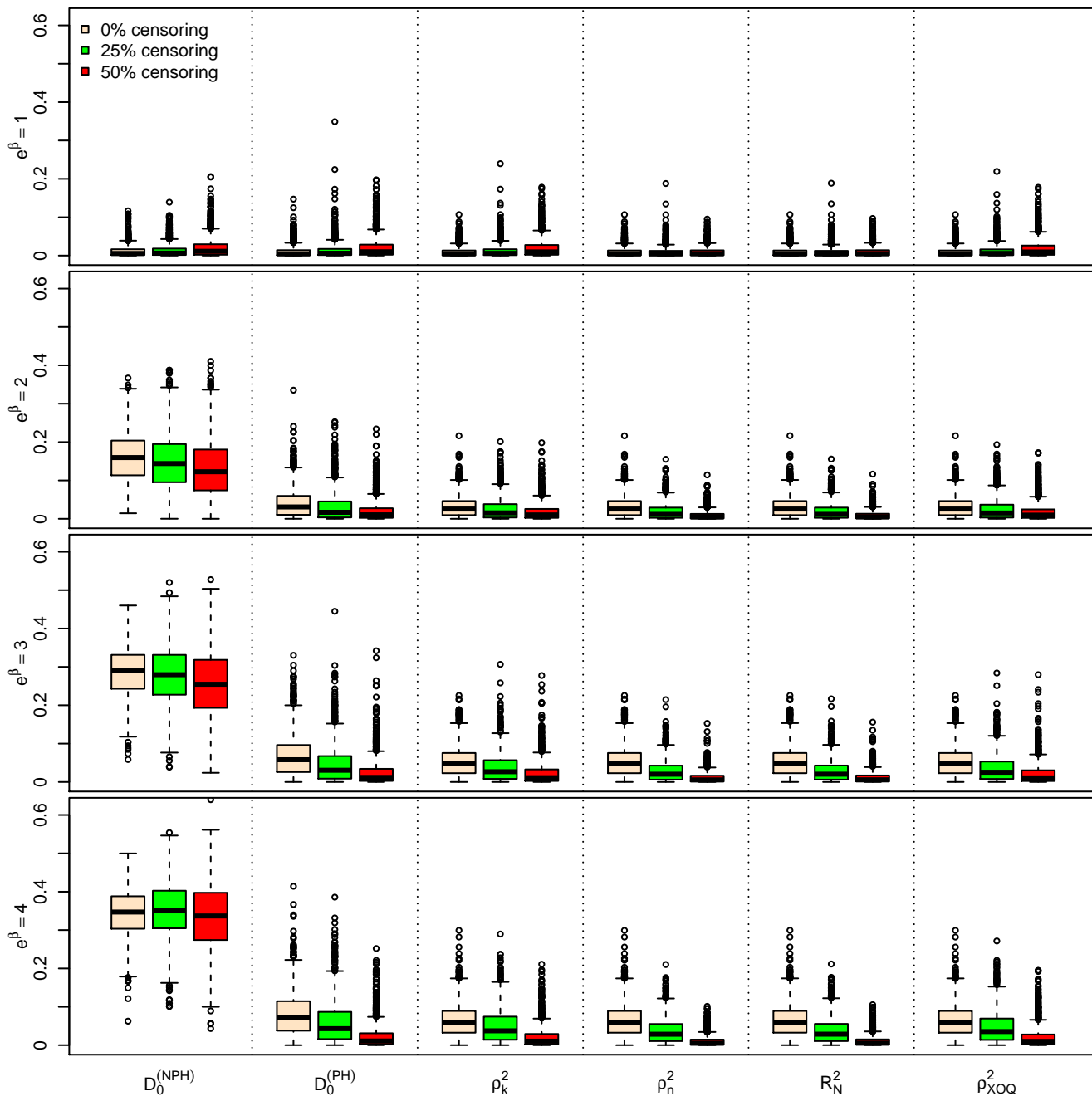

Supplement: Additional file 5 — Simulations results for , , and , for n = 100 subjects, and a uniform censoring (1,000 repetitions). Graphic: Boxplots of the different indices according to the values of eβ and pc. [file 1471-2288-11-28-S5.PDF]

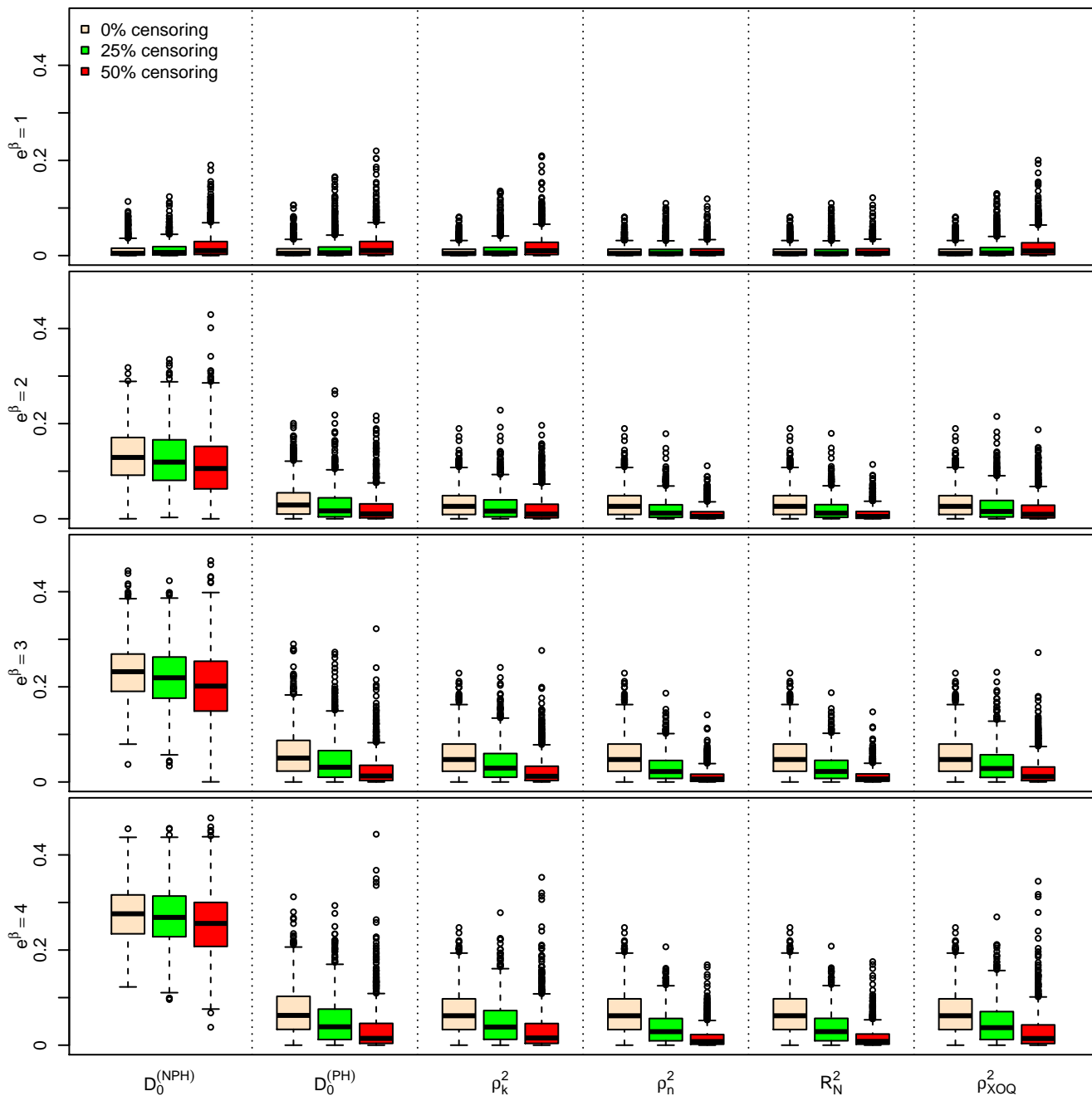

Supplement: Additional file 6 — Simulations results for , , and , for n = 100 subjects, and a uniform censoring (1,000 repetitions). Graphic: Boxplots of the different indices according to the values of eβ and pc. [file 1471-2288-11-28-S6.PDF]

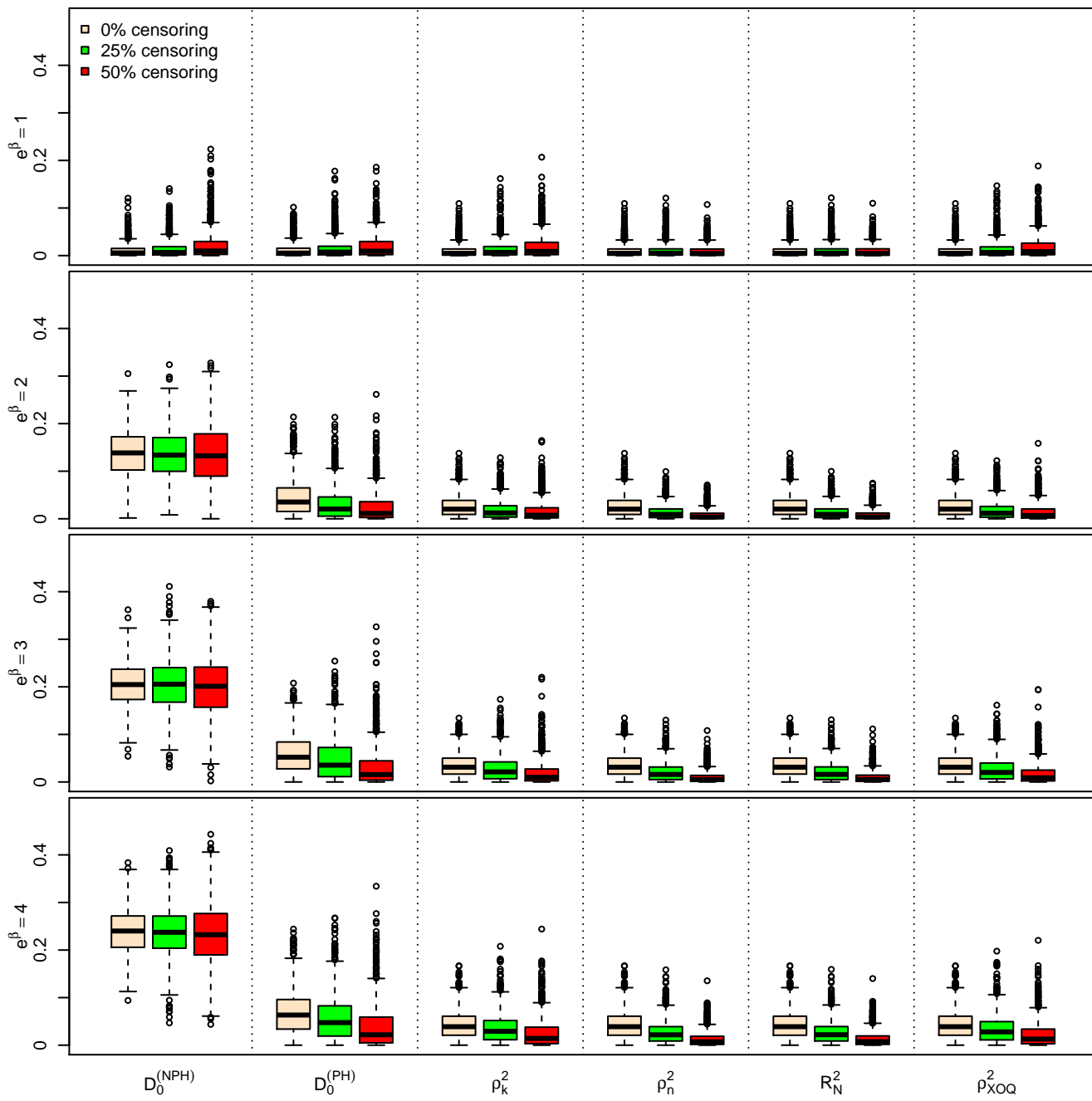

Supplement: Additional file 7 — Simulations results for , , and , for n = 100 subjects, and a uniform censoring (1,000 repetitions). Graphic: Boxplots of the different indices according to the values of eβ and pc. [file 1471-2288-11-28-S7.PDF]
