# Supplementary material for: A pseudo-R2 measure for selecting genomic markers with crossing hazards functions
Source: BMC Med Res Methodol. 2011 Mar 15;11:28. doi: 10.1186/1471-2288-11-28 (PMC3068986; doi:10.1186/1471-2288-11-28)
Supplement: Additional file 8 — Biological processes (obtained from the PANTHER classification system) for the lung cancer study cohort. Table: List of the biological process obtained according to and . [file 1471-2288-11-28-S8.PDF]

Additional file 8 - Biological processes (obtained from the PANTHER classification system) for the lung cancer study

Table: Biological processes for the lung cancer study according to (a) $\mathbf{D}_0^{(\mathrm{NPH})}$ and to (b) $\mathbf{D}_0^{(\mathrm{PH})}$

Biological process	♯ Genes NCBI	♯ observed	♯ expected	P-value
**(a) Selection with $\mathbf{D}_0^{(\mathbf{NPH})}$**				
nucleobase, nucleoside, nucleotide and nucleic acid metabolic process	3825	53	30.74	$2.17 \cdot 10^{-5}$
metabolic process	8267	86	66.43	$1.20 \cdot 10^{-3}$
primary metabolic process	7950	83	63.88	$1.47 \cdot 10^{-3}$
cell cycle	1840	23	14.79	$2.25 \cdot 10^{-2}$
polyphosphate catabolic process	4	1	0.03	$3.16 \cdot 10^{-2}$
cellular component organization	1443	18	11.6	$4.24 \cdot 10^{-2}$
vesicle-mediated transport	1160	15	9.32	$4.75 \cdot 10^{-2}$
**(b) Selection with $\mathbf{D}_0^{(\mathbf{PH})}$**				
oxidative phosphorylation	76	3	0.57	$2.02 \cdot 10^{-2}$
intracellular signaling cascade	1568	19	11.81	$2.72 \cdot 10^{-2}$
lipid metabolic process	1119	3	8.43	$2.83 \cdot 10^{-2}$
muscle contraction	448	0	3.38	$3.29 \cdot 10^{-2}$
spermatogenesis	501	8	3.77	$3.68 \cdot 10^{-2}$
