# Supplementary material for: A pseudo-R2 measure for selecting genomic markers with crossing hazards functions
Source: BMC Med Res Methodol. 2011 Mar 15;11:28. doi: 10.1186/1471-2288-11-28 (PMC3068986; doi:10.1186/1471-2288-11-28)
Supplement: Additional file 9 — Lists of the cell cycle related transcripts among the selection according to the value of the index calculated either under the crossing hazards effect or the PH model. Tables: Lists of the cell cycle related transcripts according to and . [file 1471-2288-11-28-S9.PDF]

Additional file 9: Lists of the cell cycle related transcripts among the selection according to the value of the index calculated either under the crossing hazard effect or the PH model.

Table 1: Cell-cycle related genes selected according to $\mathbf{D}_0^{(\mathrm{NPH})}$

AffyID	Gene Symbol	UniGene Name	Cytoband	$\mathbf{D}_0^{(\mathrm{NPH})}$
221326-s-at	*TUBD1*	tubulin, delta 1	chr17q23.1	37.9%
209661-at	*KIFC3*	kinesin family member C3	chr16q13-q21	35.9%
208228-s-at	*FGFR2*	fibroblast growth factor receptor 2	chr10q26	34.5%
206003-at	*CEP135*	centrosomal protein 135kDa	chr4q12	34.4%
225237-s-at	*MSI2*	musashi homolog 2 (Drosophila)	chr17q22	34.1%
1562139-a-at	*FOXP2*	forkhead box P2	chr7q31	34.0%
206113-s-at	*RAB5A*	RAB5A, member RAS oncogene family	chr3p24-p22	33.7%
200796-s-at	*MCL1*	myeloid cell leukemia sequence 1 (BCL2-related)	chr1q21	32.9%
221281-at	*SRC*	v-src sarcoma (Schmidt-Ruppin A-2) viral oncogene homolog (avian)	chr20q12-q13	32.7%
207319-s-at	*CDC2L5*	cell division cycle 2-like 5 (cholinesterase-related cell division controller)	chr7p13	32.5%
213023-at	*UTRN*	utrophin	chr6q24	32.1%
214908-s-at	*TRRAP*	transformation/transcription domain-associated protein	chr7q21.2-q22.1	32.1%
222563-s-at	*TNKS2*	tankyrase, TRF1-interacting ankyrin-related ADP-ribose polymerase 2	chr10q23.3	32.1%
1555346-at	*CDC20B*	cell division cycle 20 homolog B (S. cerevisiae)	chr5q11.2	31.9%
219944-at	*CLIP4*	CAP-GLY domain containing linker protein family, member 4	chr2p23.2	31.8%
239223-s-at	*FBXL20*	F-box and leucine-rich repeat protein 20	chr17q12	31.6%
222540-s-at	*RSF1*	remodeling and spacing factor 1	chr11q14.1	31.1%
203639-s-at	*FGFR2*	fibroblast growth factor receptor 2	chr10q26	31.0%
224010-at	*ANAPC11*	anaphase promoting complex subunit 11	chr17q25.3	31.0%
215739-s-at	*TUBGCP3*	tubulin, gamma complex associated protein 3	chr13q34	30.9%
206235-at	*LIG4*	ligase IV, DNA, ATP-dependent	chr13q33-q34	30.8%
243999-at	*SLFN5*	schlafen family member 5	chr17q12	30.5%
211103-at	*MYO7A*	myosin VIIA	chr11q13.5	30.4%
211401-s-at	*FGFR2*	fibroblast growth factor receptor 2	chr10q26	30.1%
212308-at	*CLASP2*	cytoplasmic linker associated protein 2	chr3p22.3	29.8%

Table 2: Cell-cycle related genes selected according to $\mathbf{D}_0^{(\mathrm{PH})}$

AffyID	Gene Symbol	UniGene Name	Cytoband	$\mathbf{D}_0^{(\mathrm{PH})}$
226765-at	*SPTBN1*	CDNA clone IMAGE:3897439	chr2p21	41.0%
1566851-at	*TRIM42*	tripartite motif-containing 42	chr3q23	37.1%
202257-s-at	*CD2BP2*	CD2 (cytoplasmic tail) binding protein 2	chr16p11.2	36.3%
203348-s-at	*ETV5*	ets variant 5	chr3q28	35.8%
210356-x-at	*MS4A1*	membrane-spanning 4-domains, subfamily A, member 1	chr11q12	34.3%
205861-at	*SPIB*	Spi-B transcription factor (Spi-1/PU.1 related)	chr19q13.3-q13.4	34.2%
233251-at	*STRBP*	Chromosome 9 open reading frame 45, mRNA	chr9q33.3	31.5%
216609-at	*TXN*	Full length insert cDNA clone YI46D09	chr9q31	31.4%
214114-x-at	*FASTK*	Fas-activated serine/threonine kinase	chr7q35	30.9%
202252-at	*RAB13*	RAB13, member RAS oncogene family	chr1q21.2	30.9%
202810-at	*DRG1*	developmentally regulated GTP binding protein 1	chr22q12.2	30.7%
202676-x-at	*FASTK*	Fas-activated serine/threonine kinase	chr7q35	30.7%
229664-at	*MAPK8*	mitogen-activated protein kinase 8	chr10q11.22	30.5%
224434-s-at	*MORG1*	mitogen-activated protein kinase organizer 1	chr19p13.13	30.3%
210187-at	*FKBP1A*	FK506 binding protein 1A, 12kDa	chr20p13	29.9%
210111-s-at	*KLHDC10*	KIAA0265 gene	chr7q32.2	29.7%
